# 

**Published:** 1878-11

**Authors:** 


### CONTENTS:

ARTICLE I.—Hard Rubber Appliances for Congenital Cleft Palate. By Thomas Brian Gunning, D. D. S., M. D. .... 289

ARTICLE II.—Maryland and District of Columbia Dental Association .... 298

ARTICLE III.—Teeth and Flour. By Ephraim Cutter, M. D .... 311

ARTICLE IV.—Dentition as one of the Causes of Cholera Infantum. By J. H. Nowlin, M. D. .... 316

ARTICLE V.—Some Experience with Dental Amalgams. By H. S. Chase, D. D. S., M. D. 320

ARTICLE VI.—The Action of Anæsthetics .... 323

ARTICLE VII.—How to Make Nerve Bristles. By Theo. F. Chupein, D. D. S. .... 327

ARTICLE VIII.—Treatment of Periostitis. By Dr. A. M. Holmes .... 328

ARTICLE IX.—Physiology of the Lymphatic System. By Z. C. McElroy, M. D. .... 329

### EDITORIAL, ETC.

Maryland and District of Columbia Dental Association .... 330

### OBITUARY.

Philip H. Austen, M. D., D. D. S .... 332

Charles Knower, D. D. S., M. D .... 333

### BIBLIOGRAPHICAL.

The Physician's Visiting List for 1879 .... 334

Urethral Stricture. By Thos. R. Brown, M. D .... 334

The Intra-Venous Injection of Milk. By T. Gaillard Thomas, M. D .... 334

### MONTHLY SUMMARY.

Chloroform—Rules for its Administration, Etc .... 335

A Peculiar Mouth Affection .... 336

Nitrite of Amyl and Chloroform as an Anæsthetic .... 336

The Bael as an Astringent .... 336

